# Antiviral Characterization of Advanced Materials: Use of Bacteriophage Phi 6 as Surrogate of Enveloped Viruses Such as SARS-CoV-2

**DOI:** 10.3390/ijms23105335

**Published:** 2022-05-10

**Authors:** Ángel Serrano-Aroca

**Affiliations:** Biomaterials and Bioengineering Lab, Centro de Investigación Traslacional San Alberto Magno, Universidad Católica de Valencia San Vicente Mártir, c/Guillem de Castro 94, 46001 Valencia, Spain; angel.serrano@ucv.es

**Keywords:** bacteriophage phi 6, SARS-CoV-2, biosafety conditions, antiviral materials, antiviral characterization, coatings, nanomaterials, composites, films, extracts, porous supports

## Abstract

The bacteriophage phi 6 is a virus that belongs to a different Baltimore group than SARS-CoV-2 (group III instead of IV). However, it has a round-like shape and a lipid envelope like SARS-CoV-2, which render it very useful to be used as a surrogate of this infectious pathogen for biosafety reasons. Thus, recent antiviral studies have demonstrated that antiviral materials such as calcium alginate hydrogels, polyester-based fabrics coated with benzalkonium chloride (BAK), polyethylene terephthalate (PET) coated with BAK and polyester-based fabrics coated with cranberry extracts or solidified hand soap produce similar log reductions in viral titers of both types of enveloped viruses after similar viral contact times. Therefore, researchers with no access to biosafety level 3 facilities can perform antiviral tests of a broad range of biomaterials, composites, nanomaterials, nanocomposites, coatings and compounds against the bacteriophage phi 6 as a biosafe viral model of SARS-CoV-2. In fact, this bacteriophage has been used as a surrogate of SARS-CoV-2 to test a broad range of antiviral materials and compounds of different chemical natures (polymers, metals, alloys, ceramics, composites, etc.) and forms (films, coatings, nanomaterials, extracts, porous supports produced by additive manufacturing, etc.) during the current pandemic. Furthermore, this biosafe viral model has also been used as a surrogate of SARS-CoV-2 and other highly pathogenic enveloped viruses such as Ebola and influenza in a wide range of biotechnological applications.

## 1. Introduction

The dramatic spread of severe acute respiratory syndrome coronavirus 2 (SARS-CoV-2), which caused the coronavirus disease (COVID-19) pandemic, has made many researchers focus their attention on the development of new antiviral materials, coatings and novel compounds capable of inactivating this pathogen [1,2,3,4]. Thus, much progress has been achieved in antiviral materials research to develop, for example, anti-SARS-CoV-2 biodegradable polymers such as alginate films [5] or a chitosan-based nasal spray [6]. Several ceramic and metallic materials, such as silicon nitride bioceramic and aluminum, have also shown antiviral activity against this pathogen [7,8]. Nanomaterials made of silver, copper or carbon, among others, have also shown anti-SARS-CoV-2 capacity [1,9]. Furthermore, new coatings, among many other material engineering approaches, have shown intrinsic antiviral capacity, which is very promising to combat COVID-19 and other diseases caused by enveloped viruses [4,10,11].

Antiviral research on this highly infectious enveloped virus requires having access to biosafety level 3 (BSL-3) labs. However, since there is a shortage of this type of lab worldwide, and most of them are often very busy focusing their research on the development of vaccines, new drugs and drug repositioning, it is very difficult for many researchers working in this field to characterize the antiviral properties of new materials. In this context, the bacteriophage phi 6 is a virus that belongs to group III of the Baltimore classification instead of group IV like SARS-CoV-2. However, it has a round-like shape and a lipid envelop like SARS-CoV-2. Since the antiviral mechanism of action is often associated with disruption of the viral membrane of the lipid envelope [1,2,3,4], this viral model can be successfully used as a surrogate of SARS-CoV-2 in biosafety conditions. Thus, many recent antiviral studies have used this viral model to study the anti-SARS-CoV-2 properties of a broad range of materials, coatings and compounds of different chemical natures (polymers, metals, alloys, ceramics, composites, etc.) and forms (films, coatings, nanomaterials, extracts, porous supports produced by additive manufacturing, etc.) during the current pandemic [12,13,14,15,16,17,18,19]. Furthermore, a broad range of materials, coatings and compounds have been tested against both the bacteriophage phi 6 and SARS-CoV-2 to validate this biosafe viral model [5,10,11,20,21].

The bacteriophage phi 6 has been used in a wide range of biotechnological applications as a valuable surrogate virus of SARS-CoV-2 [22,23,24,25,26] and as a surrogate of other highly pathogenic enveloped viruses such as influenza and Ebola [27,28,29]. Therefore, researchers with no access to BSL-3 facilities can characterize the antiviral properties of a broad range of materials, nanomaterials, coatings and compounds of different chemical natures and forms using the bacteriophage phi 6 as a surrogate of SARS-CoV-2 or other enveloped viruses such as Ebola and influenza.

## 2. SARS-CoV-2 and Bacteriophage Phi 6

**Figure 1:**
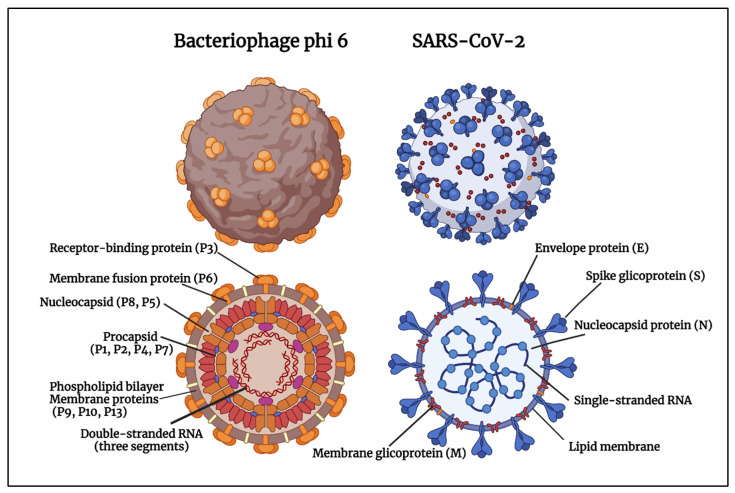
Bacteriophage phi 6 and SARS-CoV-2. Created by Ángel Serrano-Aroca with Biorender.

SARS-CoV-2 is the seventh human coronavirus [30,31] and can spread much faster than SARS-CoV and MERS-CoV [32,33,34,35,36,37,38,39], especially the recent Omicron variant of concern [40,41]. SARS-CoV-2 is a positive-sense single-stranded RNA virus with a lipid envelope [42] (Baltimore group IV [43]). However, the bacteriophage phi 6 has a three-part, segmented genome, with a 13.5 kb-long double-stranded RNA virus (Baltimore group III [43]), but it also has a lipid envelope that protects the internal nucleocapsid structure [44] like SARS-CoV-2 (Figure 1).

Figure 1 shows how the enveloped bacteriophage phi 6 has a phospholipid bilayer that contains the P9, P10, P13 and P6 membrane proteins, and the receptor-binding protein P3 that forms the outermost layer of the particle [45]. It contains a procapsid composed of three segments of the double-stranded RNA viral genome and the P1, P2, P4 and P7 proteins. The procapsid and a shell of the P8 and P5 proteins form the nucleocapsid. SARS-CoV-2 is another enveloped virus with single-stranded RNA, spike glycoproteins (S), nucleocapsid proteins (N), envelope proteins (E), membrane glycoproteins and a lipid membrane [46,47]. The size of the bacteriophage phi 6 is about 85 nm [48]. The size of SAR-CoV-2 particles ranges from 60 to 140 nm according to Zu et al. [49], or between 70 nm and 110 nm [50], and an average diameter of about 76 nm was reported in another study [51].

**Figure 2:**
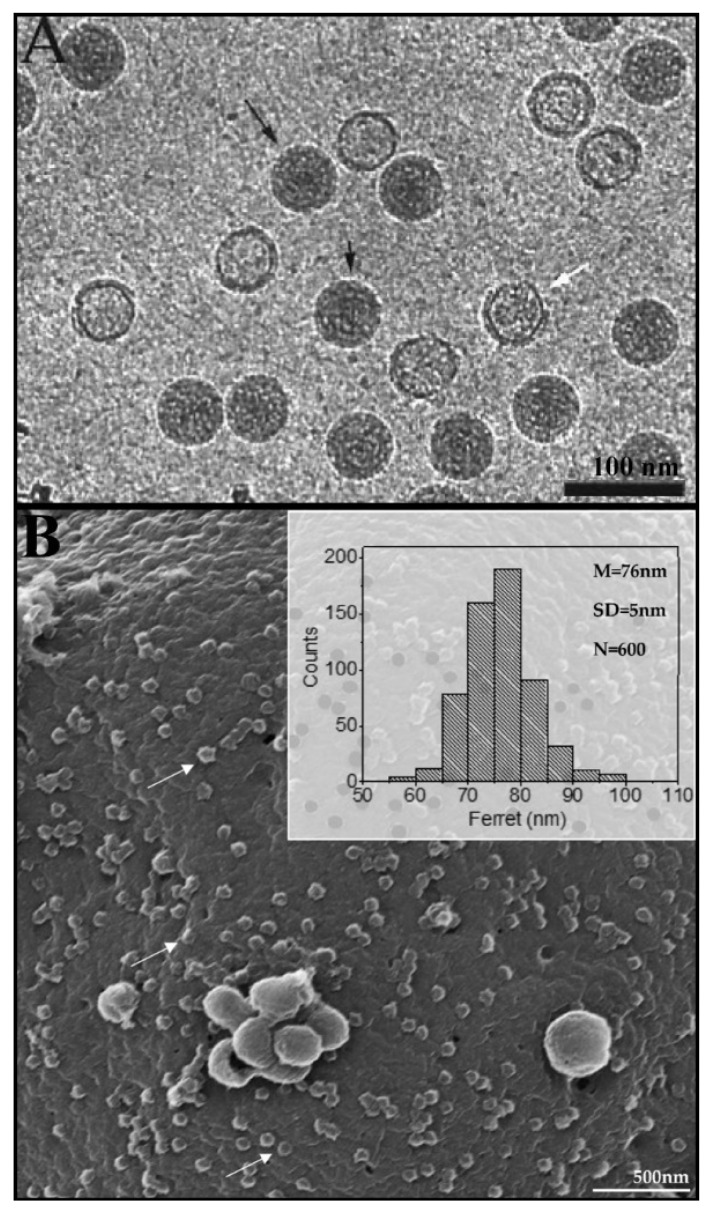
Microscopic images of the bacteriophage phi 6 and SARS-CoV-2: (**A**) Cryo-electron microscopy image of the nucleocapsids (NCs) of the bacteriophage phi 6 highlighted with black arrows. A partially disrupted NC is pointed out by a white arrow, where the core can be appreciated with a clear angular inner layer. Reprinted in part with permission from [52]. Copyright 1997 JOHN WILEY AND SONS. (**B**) Diameter of SARS-CoV-2 viral particles attached in cell membrane (white arrow) (helium ion microscopy image). The number of measured particles (N), mean (M), standard deviation (SD) and virus particles (arrow) are indicated [51].

Both viruses have a round-like shape as shown in Figure 1 and Figure 2 by advanced microscopic techniques.

## 3. Validation of Bacteriophage Phi 6 as Viral Model of SARS-CoV-2

The emergence of new SARS-CoV-2 variants such as Delta and Omicron has proved the urgent need for antiviral research, in which bacteriophages may help significantly [53]. Many new and commonly used antimicrobial compounds have been tested against SARS-CoV-2 during the current pandemic. Thus, benzalkonium chloride (BAK) (0.1%) and hand soap (1:49) have shown in vitro virucidal activity against SARS-CoV-2 [54]. BAK is extensively used as a key compound of many household disinfecting wipes and sprays and is also employed as an additive in many soaps and non-alcoholic hand sanitizers [55,56,57]. The use of hand soap for hand washing has been recommended to prevent COVID-19 transmission by the Centers for Disease Control and Prevention since the beginning of the pandemic [58]. Furthermore, biobased products such as cranberry extracts have also shown potent antiviral capacity against enveloped viruses such as the herpes simplex virus types 1 and 2 (HSV-1 and HSV-2) [59] and influenza virus (IFV) [60]. Therefore, new composite materials developed with biofunctional coatings of BAK, cranberry extracts or solidified hand soap have shown antiviral activity against both the bacteriophage phi 6 and SARS-CoV-2 at even very short viral contact times (Table 1).

**Figure 3:**
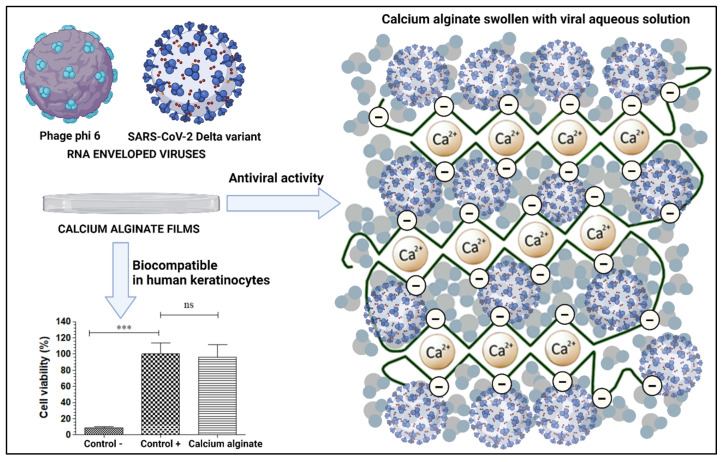
Antiviral properties of biocompatible calcium alginate films against enveloped viruses such as the bacteriophage phi 6 and SAS-CoV-2. Calcium alginate swollen structure in viral aqueous solution. Cell viability results in human keratinocytes after performing ANOVA with subsequent Tukey’s post hoc test: *** *p* > 0.001; ns, not significant [5].

Therefore, these antiviral studies validate this biosafe viral model as a very useful surrogate of SARS-CoV-2 for these types of materials (Table 1). Table 1 shows very similar percentages of inactivation of both types of viruses after the same low viral contact times. The antiviral activity is time-dependent and thus it increases with increasing viral contact time. Inactivation on antiviral surfaces is desired to occur at low viral contact times. For example, biocompatible films of calcium alginate recently showed intrinsic high inactivation capacity against the bacteriophage phi 6 and the SARS-CoV-2 Delta variant after 30 min of viral contact (Figure 3) [5].

These antiviral tests showed a 1.43-log reduction (94.92% viral inactivation) for the bacteriophage phi 6 and a 1.64-log reduction (96.94% viral inactivation) for SARS-CoV-2 after 30 min of viral contact (see Table 1). The antiviral mechanism of the calcium alginate films can be attributed to their compacted negative charges that may bind to viral envelopes, inactivating membrane receptors [5]. Another material with even higher antiviral capacity consists of a non-woven fabric with BAK produced by the dip coating method [61]. This composite fabric showed potent antiviral activity against the bacteriophage phi 6 and SARS-CoV-2 (100% and 99.75% of viral inhibition after just 1 min of viral contact, respectively) [11]. This antiviral technology applied to commercial non-woven fabrics is also capable of inactivating life-threatening multidrug-resistant pathogens such as methicillin-resistant *Staphylococcus aureus* (MRSA) and methicillin-resistant *Staphylococcus epidermidis* (MRSE). Therefore, it can also be used to combat bacterial resistance to antibiotics, which has become a real threat to humanity. In fact, the World Health Organization (WHO) has predicted that more people could die from multidrug-resistant pathogens than from cancer by the year 2050 [62].

**Figure 4:**
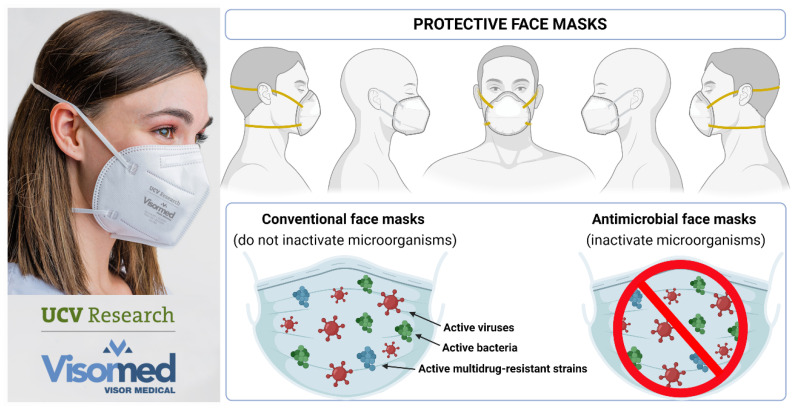
Antimicrobial face mask (FFPCOVID MASK) that inactivates enveloped viruses such as the bacteriophage phi 6 and SARS-CoV-2, and MRSA and MRSE multidrug-resistant bacteria, from UCV Research-Visormed [63] (**left**); protective face masks: difference between conventional face masks and antimicrobial face masks (**right**). Created by Ángel Serrano-Aroca with Biorender.

Therefore, due to the excellent properties of this fabric made of a smart material capable of combating antibiotic-resistant bacteria, COVID-19 and other viral diseases caused by enveloped viruses, this technology was transferred from lab to industry, and more specifically from the Laboratory of Biomaterials and Bioengineering at Centro de Investigación Traslacional San Alberto Magno at the Universidad Católica de Valencia San Vicente Mártir, the Serrano BBlab (www.serranobblab.com, Valencia, Spain, accessed on 19 March 2022), to the Visormed company (https://visormed.com/es, Alicante, Spain, accessed on 19 March 2022) during the current pandemic for the fabrication of antimicrobial face masks on a large scale [63] (Figure 4, left).

These were the first advanced face masks with antimicrobial properties against enveloped viruses such as SARS-CoV-2 and multidrug-resistant bacteria reported in the literature and produced on an industrial scale in the world. These next-generation antimicrobial masks will be very useful for the current and future pandemics, and they constitute a very valuable preventive tool against the increasing microbial resistance to antibiotics. The use of the bacteriophage phi 6 as a surrogate of enveloped respiratory viruses such as SARS-CoV-2 or influenza in the development of new antimicrobial face masks has helped significantly in accelerating the scientific progress in this field [3], and the FFCOVID MASK produced by UCV Research-Visormed is a clear example (Figure 4, right).

**Figure 5:**
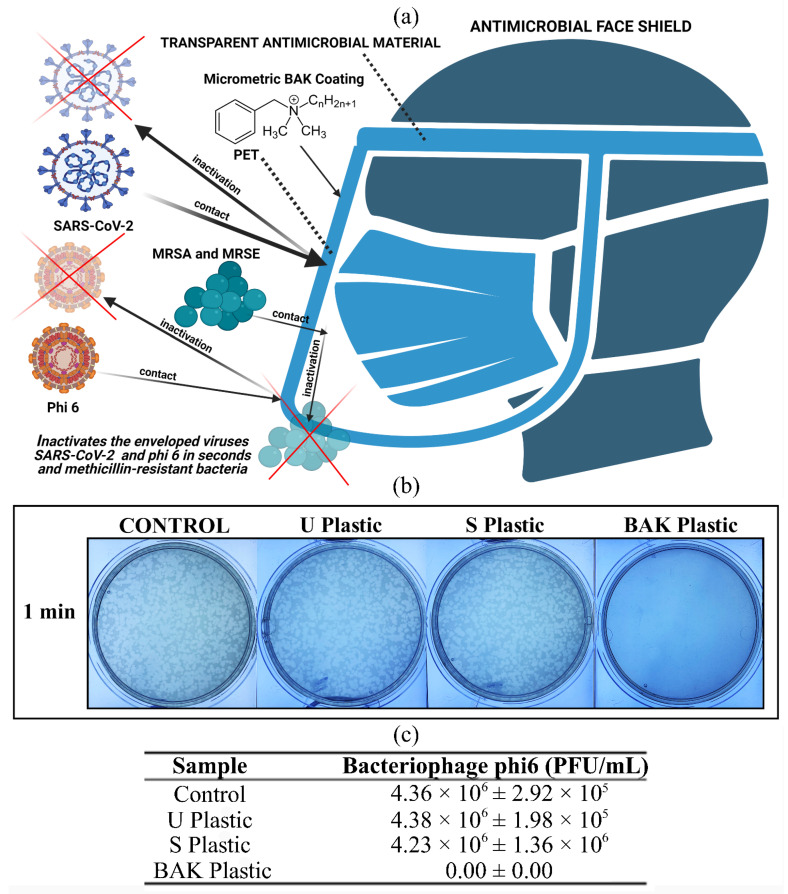
Antiviral characterization of an antimicrobial face shield using the bacteriophage phi 6 as a viral model of SARS-CoV-2 for biosafety reasons: (**a**) Antimicrobial face shield developed by the Serrano BBlab (www.serranobblab.com, accessed on 19 March 2022): next generation of preventive equipment against infections caused by enveloped viruses such as SARS-CoV-2 and multidrug-resistant bacteria. The material is composed of polyethylene terephthalate (PET) coated with benzalkonium chloride (BAK). The double-layer method was used to determine the loss of viral viability after 1 min of viral contact: (**b**) Bacteriophage phi 6 titration images of undiluted samples for the materials. The reduction in infection capacity can be observed by the reduction in white spots. (**c**) Decrease in infection titers expressed in plaque-forming units per mL (PFU/mL). CONTROL: bacteriophages without being in contact with any material; U Plastic: untreated PET; S plastic: PET treated with solvent; BAK plastic: PET treated with solvent and BAK [20].

Next-generation antimicrobial face shields have also been developed by the same lab by dip coating transparent PET sheets with a BAK solution (Figure 5).

This antimicrobial face shield showed potent antiviral activity against the bacteriophage phi 6 and SARS-CoV-2 (100% and 90% of viral inhibition after 1 min of viral contact, respectively) [20]. This was the first face shield with antimicrobial properties against enveloped viruses such as SARS-CoV-2 and multidrug-resistant bacteria reported in the literature. The antiviral mechanism of action of BAK against enveloped viruses is attributed to the positively charged nitrogen atoms that can disrupt the viral phospholipid bilayer membrane [64], and the spike glycoproteins that interact with the ACE2 receptor in the infection of host cells [65].

**Figure 6:**
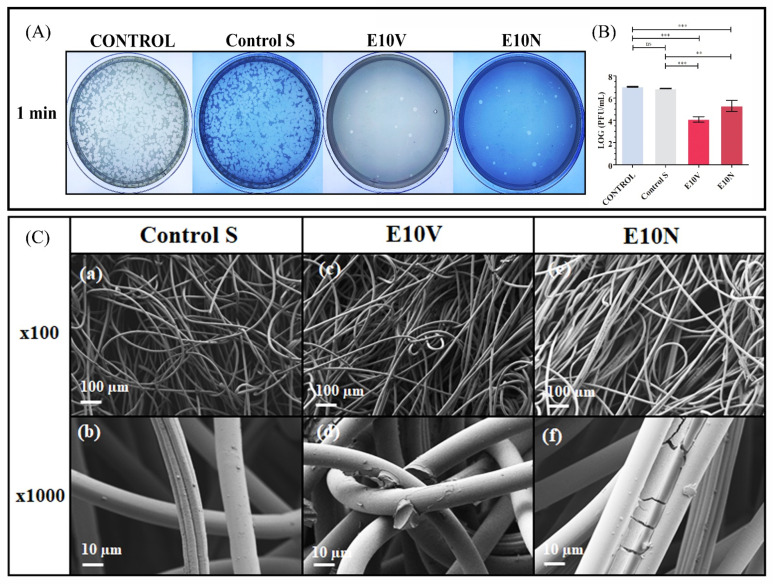
Antiviral characterization of non-woven fabrics coated with two types of commercial cranberry extracts against the bacteriophage phi 6 for biosafety reasons: (**A**) The double-layer method was used to determine the viral viability after 1 min of viral contact (titration images of undiluted samples). These images show the reduction in infection capacity (reduction in white spots). (**B**) Reduction in infection titers of the bacteriophage phi 6 in a logarithm of plaque-forming units per mL (log(PFU/mL)) measured by the double-layer method at 1 min of viral contact. Statistical analysis: *** *p* > 0.001; ** *p* > 0.01; ns: not significant. (**C**) High-resolution field-emission scanning electron microscopy (HR-FESEM) of the non-woven fabrics, at two different magnifications (×100 and ×1000), before (**a**,**b**) and after the treatment with the VITAFAIR cranberry extract (E10V) (**c**,**d**) or the NUTRIBIOLITE cranberry extract (E10N) (**e**,**f**). CONTROL: bacteriophages without being in contact with any material; Control S: uncoated non-woven fabric [10].

Another developed biobased technology consists of dip coating non-woven fabrics with two types of commercial cranberry extracts (see Table 1 and Figure 6) [10].

These antiviral fabrics showed more than 99% of viral inactivation against SARS-CoV-2 and the bacteriophage phi 6 after 1 min of viral contact [10]. Cranberry extracts possess antiviral properties as they contain antimicrobial A-type proanthocyanidins (PACs) that cause alterations of the viral envelope glycoproteins [10,59,60]. These next-generation fabrics fabricated with biobased coatings have also shown antibacterial activity against MRSA and MRSE multidrug-resistant bacteria [10].

Another antiviral strategy developed to provide low-cost antiviral face masks for potential use in developed and undeveloped countries for the current pandemic consists of producing non-woven fabrics coated with solidified hand soap [21]. This low-cost technology provides strong viral inactivation capacity against the bacteriophage phi 6 and SARS-CoV-2 (100% and 98% after 1 min of viral contact, respectively) to non-woven fabrics. Furthermore, face masks fabricated with these antiviral non-woven fabrics did not show any toxic effect on human keratinocytes [21].

The antiviral mechanism of action against both types of viruses, namely, the bacteriophage phi 6 and SARS-CoV-2, is often attributed to the binding of negative [5] or positive charges [64] to viral envelopes, producing potent viral inactivation [1,2,3,4]. Both viruses are RNA viruses and have a viral envelope. However, the bacteriophage phi 6 is a double-stranded RNA virus (Baltimore group III), and SARS-CoV-2 is a single-stranded RNA virus (Baltimore group III). Therefore, they present a different genome organization, viral infectivity and replication strategy. Nonetheless, the materials presented in this section have been tested against both types of viruses, showing very similar antiviral results. Therefore, these results validate the use of this biosafe viral model of SARS-CoV-2 for these types of materials and demonstrate its promising use in antiviral materials science. However, after determining the optimal antibacteriophage phi 6 conditions, it is always recommended to test the materials against SARS-CoV-2 or alternative surrogates, such as human coronavirus 229E or murine hepatitis virus, which are approved to justify claims against COVID-19 depending on the regulatory authorities of each country [66].

## 4. Use of Bacteriophage Phi 6 for the Antiviral Characterization of Advanced Materials

Additive manufacturing (AM) is at the forefront of enabling redistributed manufacturing, which is critical in reducing the carbon footprint and enabling smart manufacturing approaches of the future [67]. In this field, the antiviral properties of a copper-tungsten-silver porous alloy filter produced by AM have also been characterized using the bacteriophage phi 6 as a biosafe viral model of SARS-CoV-2 [12] (see Table 2).

**Figure 7:**
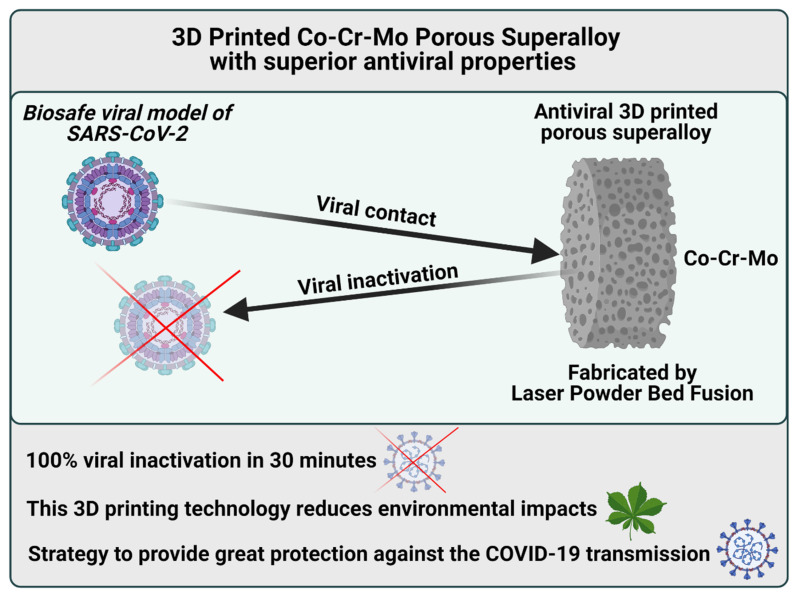
Cobalt-chromium-molybdenum porous superalloy with superior antiviral activity fabricated by additive manufacturing. Antiviral filters were tested using the bacteriophage phi 6 as a surrogate of SARS-CoV-2 for biosafety reasons [13].

In the same research line, the antiviral properties of a porous metallic cobalt-chromium-molybdenum superalloy filter produced by AM showed superior antiviral activity against the bacteriophage phi 6 as a surrogate of SARS-CoV-2 [13] (Figure 7 and Table 2).

Coatings of polyethylene (PE) packaging based on nanoparticles of ZnO and nanoparticles supplemented with carvacrol and geraniol have been tested against the bacteriophage phi 6 as a viral model of SARS-CoV-2 [14]. The antiviral properties of metal salts, metal and ceramic powders using Ag and Cu ions as doping agents and newly produced ceramic and metal surfaces have also been studied against the bacteriophage phi 6 [15]. These materials produced by spark plasma sintering and/or selective laser melting exhibited potent virucidal activity and showed different surface free energies and infiltration features. PE films with a biofunctional coating composed of layers based on CO_2_ extracts of raspberry seeds, pomegranate seeds and rosemary [16] or a mixture of the three extracts obtained via cast extrusion [17] also showed antiviral activity against the bacteriophage phi 6. CO_2_ extracts are some of the most popular non-toxic, cheap and safe solvents [68]. These antiviral materials are very promising for packaging that may protect customers’ food products against microbial putrefaction and customers (hands) at the same time. The enhancement of antiviral properties by adding a chokeberry fruit additive to rape honey was demonstrated using the bacteriophage phi 6 [18]. Mixtures of *Scutellaria baicalensis* and *Glycyrrhiza L.* extracts have also shown potent antiviral activity against this bacteriophage [19]. In addition to studying the antiviral properties, it is very important to characterize the toxicity of the materials and compounds in order to ensure their safe applications for human beings. Antiviral materials that show in vitro or in vivo toxicity present low interest in antiviral research science. Thus, no toxicity tests were performed in any of the studies reported in Table 2, and further research should be performed in this direction to find safe antiviral solutions for human beings.

On the other hand, the bacteriophage phi 6 has been studied in a wide range of biotechnological applications as a valuable surrogate virus of SARS-CoV-2. Thus, the survival of viruses in evaporated saliva microdroplets deposited on glass surfaces was studied with this bacteriophage as a viral model of SARS-CoV-2 [22]. The persistence of bacteriophage phi 6 virions was studied in aquatic environments to better understand potential mechanisms that may prolong their dissemination as a viral model of SARS-CoV-2 [23]. The bacteriophage phi 6 has also been used to evaluate ultraviolet-C light for rapid decontamination of airport security bins in the era of SARS-CoV-2 [24], and to study the surface disinfection efficacy with chlorine and antimicrobial surfaces [25]. In a similar way, the effectiveness of a fully automatic room decontamination system based on ozone was assessed against the bacteriophage phi 6 as a surrogate virus for the current SARS-CoV-2 pandemic [26].

In addition, the bacteriophage phi 6 has also been used as a surrogate of other enveloped viruses such as Ebola virus [27,28], influenza virus [29], Venezuelan equine encephalitis virus [69], coronavirus SARS-CoV-1 and other pathogenic enveloped viruses [28,44,70,71,72].

Another bacteriophage, MS2, has also been used as a surrogate of SARS-CoV-2 because it is an RNA virus that belongs to the same Baltimore group, group IV [73]. However, SARS-CoV-2 is an enveloped virus, and the bacteriophage MS2 is a non-enveloped virus. It is well known that non-enveloped viruses are more resistant to inactivation than enveloped viruses [74]. Therefore, the use of phi 6 as a surrogate of SARS-CoV-2 is much more representative than the use of MS2, as it has been experimentally confirmed [25].

## 5. Conclusions

The bacteriophage phi 6 can be used as a surrogate of SARS-CoV-2 and other enveloped viruses such as Ebola and influenza for biosafety reasons. It is a virus with a round-like shape with a lipid envelope like SARS-CoV-2. Recent antiviral studies performed with both the bacteriophage phi 6 and SARS-CoV-2 have validated this biosafe viral model with a broad range of materials such as calcium alginate hydrogels and composite fabrics coated with BAK, cranberry extracts and solidified hand soap, which are used for the fabrication of antimicrobial infection prevention clothing such as next-generation face masks and antimicrobial face shields. These materials were capable of inactivating very high percentages (from 94.92 to 100%) of the bacteriophage phi 6 and SARS-CoV-2 after similar viral contact times. Therefore, antiviral tests of a broad range of biomaterials, composites, nanomaterials, nanocomposites, coatings, extracts and compounds can be performed using the bacteriophage phi 6 as a valuable biosafe viral model of SARS-CoV-2. This viral model is very useful especially for researchers with no access to biosafety level 3 facilities. In fact, this bacteriophage has been used as a surrogate of SARS-CoV-2 to test a broad range of antiviral materials and compounds of different chemical natures and forms and in a wide range of biotechnological applications during the current pandemic.

## Figures and Tables

**Table 1:** Advanced materials tested against SARS-CoV-2 and the bacteriophage phi 6: viral contact time, percentage of viral inactivation, toxicity, year and reference.

Advanced Materials	Vial Contact Time	% Viral Inactivation (Phi 6)	% Viral Inactivation (SARS-CoV-2)	Toxicity	Year	Ref.
Calcium alginate	30 min	94.92	96.94	No (human keratinocytes)	2022	[5]
Polyester/BAK	1 min	100	99.75	Not tested	2021	[11]
PET/BAK	1 min	100	90.00	Not tested	2021	[20]
Polyester/cranberry extract 1	1 min	99.89	99.91	No (*C. elegans* in vivo model)	2021	[10]
Polyester/cranberry extract 2	1 min	99.14	99.88	No (*C. elegans* in vivo model)	2021	[10]
Polyester/hand soap	1 min	100	98.00	No (human keratinocytes)	2021	[21]

**Table 2:** Advanced materials tested against the bacteriophage phi 6 as a surrogate of SARS-CoV-2: viral contact time, percentage of viral inactivation, year and reference.

Advanced Materials	Viral Contact Time	Viral Inactivation (% or Log Reduction)	Year	Ref.
3D printed copper-tungsten-silver porous alloy filter	5 h	100%	2021	[12]
3D printed cobalt-chromium-molybdenum porous superalloy filter	30 min	100%	2021	[13]
Coatings of PE based on ZnO, carvacrol and geraniol	24 h and 27 h	Not measured	2021	[14]
Metal salts, metal and ceramic powders doped with Ag and Cu ions and newly produced ceramic and metal surfaces	15 min	99.99%	2022	[15]
Polyethylene films coated with layers based on CO_2_ extracts of raspberry seeds, pomegranate seeds and/or rosemary	12 h	Up to 100%	2021	[16,17]
Addition of chokeberry fruit to rape honey	5 min	2.55-log reduction	2021	[18]
Mixtures of *Scutellaria baicalensis* and *Glycyrrhiza* L. extracts	12 h	Up to 100%	2021	[19]

## Data Availability

Data are contained within the article.
